# Circulating Tumor Cell Identification Based on Deep Learning

**DOI:** 10.3389/fonc.2022.843879

**Published:** 2022-02-16

**Authors:** Zhifeng Guo, Xiaoxi Lin, Yan Hui, Jingchun Wang, Qiuli Zhang, Fanlong Kong

**Affiliations:** Department of Oncology, Chifeng Municipal Hospital, Chifeng, China

**Keywords:** circulating tumor cells, detection, count, convolutional neural network, transfer learning

## Abstract

As a major reason for tumor metastasis, circulating tumor cell (CTC) is one of the critical biomarkers for cancer diagnosis and prognosis. On the one hand, CTC count is closely related to the prognosis of tumor patients; on the other hand, as a simple blood test with the advantages of safety, low cost and repeatability, CTC test has an important reference value in determining clinical results and studying the mechanism of drug resistance. However, the determination of CTC usually requires a big effort from pathologist and is also error-prone due to inexperience and fatigue. In this study, we developed a novel convolutional neural network (CNN) method to automatically detect CTCs in patients’ peripheral blood based on immunofluorescence *in situ* hybridization (imFISH) images. We collected the peripheral blood of 776 patients from Chifeng Municipal Hospital in China, and then used Cyttel to delete leukocytes and enrich CTCs. CTCs were identified by imFISH with CD45+, DAPI+ immunofluorescence staining and chromosome 8 centromeric probe (CEP8+). The sensitivity and specificity based on traditional CNN prediction were 95.3% and 91.7% respectively, and the sensitivity and specificity based on transfer learning were 97.2% and 94.0% respectively. The traditional CNN model and transfer learning method introduced in this paper can detect CTCs with high sensitivity, which has a certain clinical reference value for judging prognosis and diagnosing metastasis.

## Introduction

Circulating tumor cells (CTC) are all kinds of tumor cells in peripheral blood (1). Most of the CTCs undergo apoptosis or phagocytosis after entering the peripheral blood, while a minority of CTCs develop into metastasis and undergo for a period of dormancy, and lead to metastatic tumor (2, 3). Cancer recurrence and metastasis are the main causes of death in cancer patients (4, 5). A large number of experiments on esophageal squamous cell carcinoma (6), breast cancer (7, 8), prostate cancer (9) and lung cancer (10) have proved that CTCs were closely related to the prognosis of patients with advanced cancer. As a simple blood test, CTCs detection has the advantages of high safety, low cost and repeatability, which is available at any time to evaluate the prognosis and recurrence risk of patients (11, 12). Many experiments used liquid biopsy to monitor the CTCs response in patients with malignant tumors to evaluate the therapeutic response (13–15). Many studies have shown that CTCs count is closely related to prognosis, which has an important reference value for determining clinical results and recurrence risk (16–18). The fluid biopsy can predict disease progression in real time to assess tumor heterogeneity, and it was possible to detect single CTCs or cell clusters (13, 19–21). Immune enrichment with multiparameter flow cytometric is the gold standard of CTCs detection (22), but this method was limited due to the lack of tumor-specific markers, in this case, multi-label immunofluorescence staining was essential. Epithelial cell adhesion molecule (EpCAM) was often used to detect cancer cells in the blood because it mediates contact with homotype cells in epithelial tissue (23–25). The methods of CD45^+^, DNA fluorescence *in situ* hybridization (FISH) of the centromere of chromosome 8 probe (CEP8^+^)/chromosome 17 centromere duplication (CEP17^+^) have been widely used to identify CTCs (26, 27).

In recent years, rapid and automatic identification of CTCs is becoming more and more important, and the research on the automatic identification process of CTCs was also accelerating (28, 29), such as cell search system to obtain digital images (30), rare event imaging system (REIS) (31), microfluidic platform composed of multi-functional microfluidic chip and unique image processing algorithm (32). However, due to the heterogeneity of CTCs, these classification methods were often subjective. Therefore, under certain conditions, test results vary from examiner to examiner. The development of artificial intelligence (AI) has accelerated scientists’ research on machine learning. Machine learning has been widely used in medical research because of its advantages of objectivity, rapidity, and overcoming noise (33–35), especially in medical images (36, 37). As the classical algorithms of machine learning, deep learning and convolutional neural network (CNN) have made outstanding contributions in promoting medical research (38–40). Anthimopoulos et al. proposed the first problem specific deep CNN for classification of interstitial lung diseases (ILD), the results showed that (classification performance~85.5%) CNN has potential in analyzing ILD (41). Poplin et al. used the Inception-v3 neural network structure to predict potential cardiovascular risk factors in retinal fundus images (42). Le et al. constructed a deep neural network to classify Rab protein molecules through two-dimensional CNN, which provided a valuable reference for biological modeling using deep neural network (43). At present, CNN has been widely used to promote biomedical image analysis and successfully applied in cancer diagnosis and tissue identification (44, 45). Compared with the traditional machine learning methods, CNN-based automatic image processing method has the advantage of eliminating the bias caused by personal subjectivity (46). Negative enrichment combined with immuno fluorescence *in situ* hybridization (imFISH) to detect CTCs has been proven to be feasible and clinically valuable (47, 48).

In this study, we applied deep learning to identify CTCs to reduce the subjective error. ImFISH was used to detect patients’ CTCs, each image contains positive CTCs nucleus and negative control to segment the images of circulating tumor cells. CNN deep learning network was used to identify circulating tumor cells and count CTCs.

## Materials and Methods

### A Framework for Identifying CTCs

**Figure 1:**
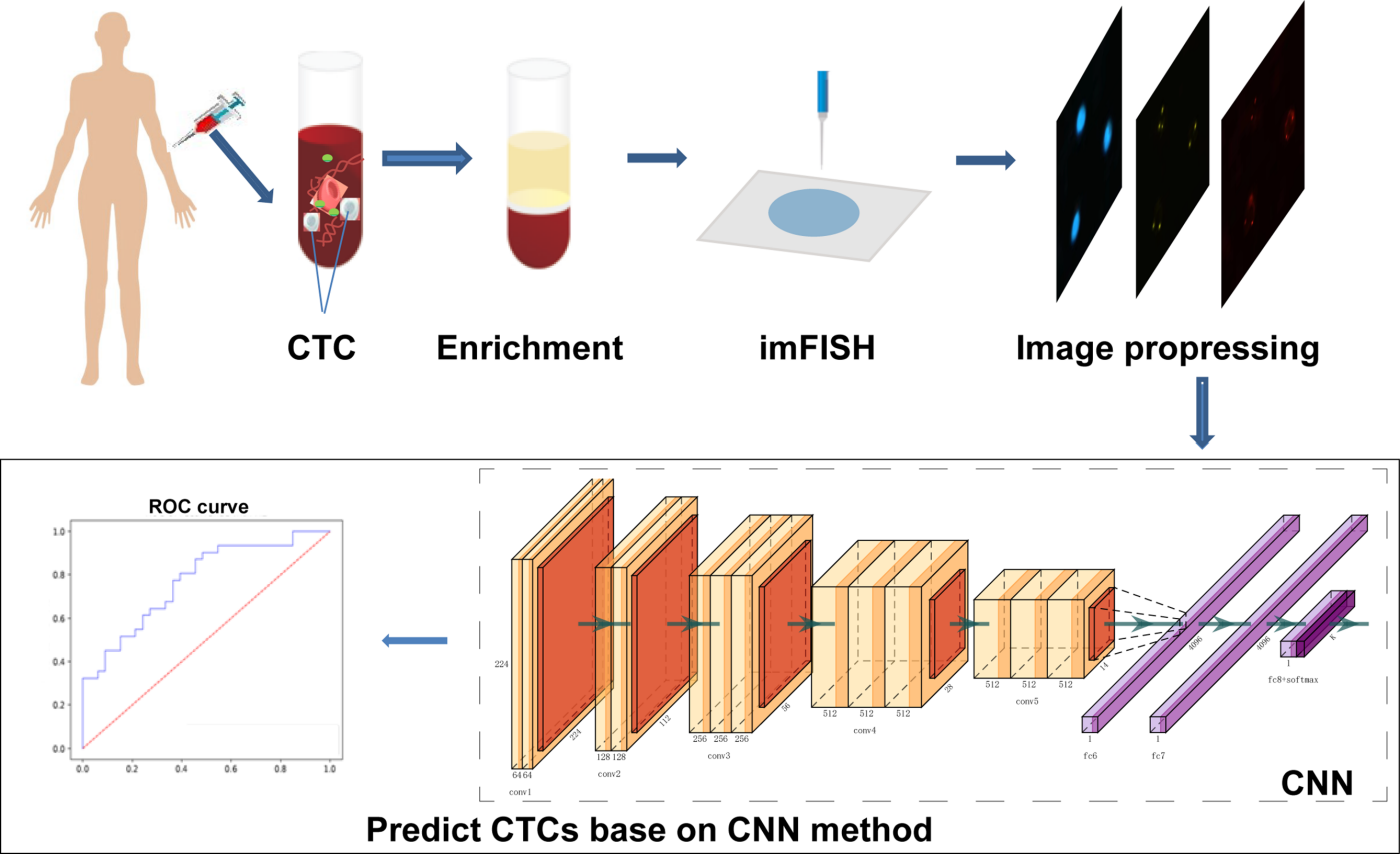
The protocol of whole process.

The complete process of identifying CTCs based on CNN was shown in **Figure 1**. Specifically, the peripheral blood of 776 cancer patients in Chifeng Municipal Hospital was collected firstly. Then the blood samples were processed by the Cyttel method. Based on the principle of immunology and with the help of magnetic particle technology, CTCs were enriched by gradually removing the components of plasma, red blood cells and white blood cells, and CTCs were processed by imFISH (26). Finally, after preprocessing the images, stratified sampling was used to divide the data, 80% of the images were used for training, and a deep learning algorithm based on CNN was used to train the model. The remaining 20% were used as separate test set. The prediction performance of the model was evaluated with the results of a 5-fold cross validation (CV).

### Samples Preparation

We conducted a retrospective study using plasma samples from the Chifeng Municipal Hospital. A total of 776 patients were enrolled from 2017 to 2019. Cancer types include lung cancer, liver cancer, gastrointestinal cancer, breast cancer, carcinoma of thyroid, NPC and others. After puncture for each patient, discarding the first 2ml blood sample to avoid skin epithelial cell pollution, then routinely collect 4ml peripheral venous blood, these samples were placed in a blood collection vessel containing acid citrate dextrose (ACD), gently inverted and mixed for 8 times before stored at room temperature, and CTCs were enriched within 24 hours after collection. The study was approved by the Ethics Committee of Chifeng Municipal Hospital.

### Enrichment of CTCs

The detection method selected in this study was Cyttel (49). The collected blood was taken out and put into a centrifuge tube for centrifugation experiment. After centrifugation at 776 g for 5 minutes, the supernatant was discarded to retain the precipitation, washed the precipitate with CS1 buffer (Cyttel Biosciences Co., Ltd., Beijing, China), and then the red blood cells were fully dissolved with CS2 buffer. Added anti-CD45^+^ monoclonal antibody binding beads and the mixture was shaken evenly for 20 minutes to fully bind with leukocytes. Another 3 ml of separation medium was added to the centrifuge tube and centrifuged at a gradient of 300 g for 5 minutes. Then the upper rare cell layer was then centrifuged at a gradient of 776 g for 5 minutes, resuspended with CS1 buffer, and the test tube was placed on a magnetic scaffold for 2 minutes. ImFISH identification was performed within 24 hours after coating, fixing, and drying.

### imFISH Identification of CTCs

The samples were fixed with a fixative, dehydrated and dried at room temperature. The slide was coated with 10μl CEP8^+^ antibody, sealed and hybridized at 37°C for 1.5 h. After hybridization, removed the cover slide and eluted the probe for 15 min. The slides were washed twice in 2×SSC. Then, the prepared CD45^+^ fluorescent antibody was added to the sample area, and the slides were placed in a humid box and incubated in an oven at 33°C for 1 hour. After incubation, fluorescent antibody against CD45^+^ was absorbed and 10 μl DAPI^+^ was added to the specimen area. Then, CTCs were observed and counted under a fluorescence microscope.

### Detection Standard of CTCs

**Figure 2:**
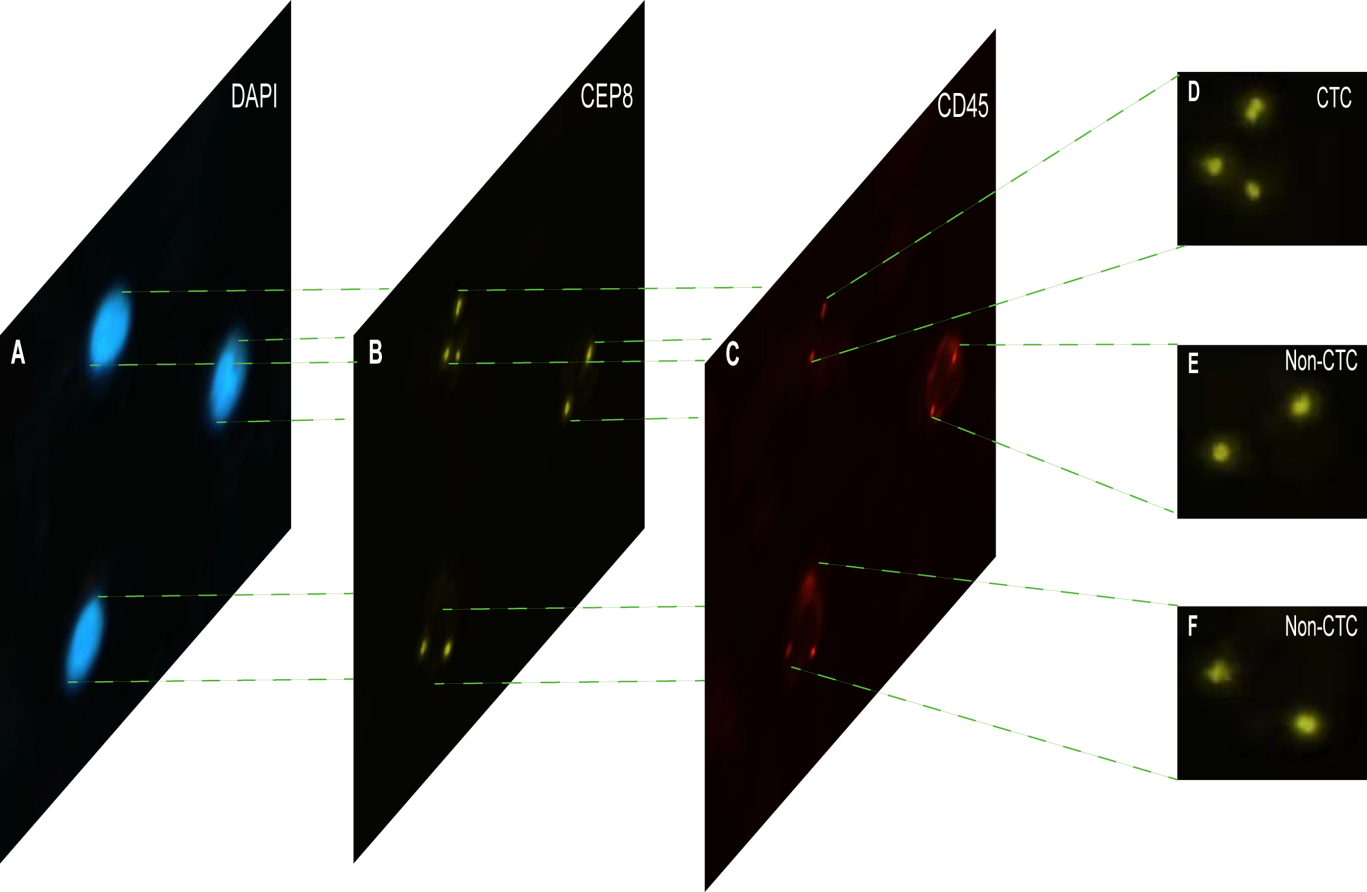
The imFISH result and CTC count results. **(A–C)** The channels (DAPI, CEP8+, CD45+) of each image by imFISH. **(D)** The cell was regarded as CTC because the number of centromeres was 3 (>2). **(E, F)** The cell was regarded as non-CTC because the number of centromeres was 2.

Each cell was divided into three different color channels: blue, orange and red. Among them, the nucleus was shown blue in DAPI^+^ (**Figure 2A**), and the centromere was shown in orange by CEP8^+^ (**Figure 2B**), and the white blood cells were stained by CD45^+^ immunofluorescence (**Figure 2C**). The interpretation criteria of CTCs count are: (1) eliminate the aggregation, superposition and interference of nuclei or impurities, (2) positive for DAPI^+^, (3) negative for CD45^+^, (4) CEP8^+^ signal points >2. That is, cells are regarded as CTCs if they are CD45-/DAPI+/CEP8≥3 (50, 51).

### Image Preprocessing

The Python package *openCV* was used to handle CTCs images (52), including color and shape conversions. To be specific, the DAPI^+^ channel was first transformed into gray scale, and then the Gaussian filter was used to denoise. After extracting the gradient of the image, the regions with a high horizontal gradient and low vertical gradient were left, and the Gaussian filter was used to denoise. Then, the blurred image was binarization, that is, each pixel was replaced by the average value of the surrounding pixels in order to smooth and replace those regions with obvious intensity changes. Due to the lack of details in the contour of the obtained image, it may interfere with the subsequent contour detection, so it is necessary to be expanded and perform four morphological corrosion and expansion respectively. After the contour of the nuclear region was found, the minimum matrix coordinates of the contour were obtained, and the coordinates were mapped to the CEP8^+^ channel and segmented.

### Computational Identification of CTC

With the development of artificial intelligence, deep learning has been widely used in medical image processing. CNN is one of the representative algorithms of deep learning, which allows higher-level feature extraction and higher-level data prediction. After images were preprocessed, stratified sampling was used to divide the data, the model was trained by 5-fold CV, and the down sampling method was used for the training set to ensure the balance of positive samples and negative samples. CTCs in a single nucleus were identified through CNN. CNN includes input layer, hidden layer and output layer, the hidden layer includes layer1, layer2 and layer3, and each layer also includes convolution layer, excitation layer and pooling layer. After the images were fed into the input layer, it first enters the first intermediate hidden layer with convolution layer is composed of 32 5x5 convolution cores, and then fed to the pool layer through the ReLU excitation layer for dimensionality reduction. After dimensionality reduction, data was output from the first hidden layer to complete the feature extraction process. Then, all features are extracted through layer2 and layer3 hidden layers in turn. Finally, it enters the output layer and outputs the result of whether it is CTC or not. The CNN in this study involved VGG16, VGG19 (53), ResNet18, ResNet50 (54) and AlexNet (55).

These pre-training models have consumed huge time resources and computing resources when developing neural networks usually. In recent years, transfer learning has become a new learning framework to solve this problem (56, 57). CNN model is pre-trained using a large number of images, and the trained model is distributed by its inventors for adoption. Transfer learning relies on the pre-trained CNN model to realize the knowledge transfer of different but related tasks, that is, using the existing knowledge learned from the completed tasks to help complete the new tasks. If the knowledge transfer is successful, it will greatly improve the learning efficiency by avoiding expensive data labeling. Transfer learning is defined as follows: a given domain *D* consists of feature space *X* and edge probability distribution *P*(*X*), a label space *y* and a prediction function *f* consist a task *T*. *D_S_* and *D_T_* represent the source domain and the target domain, respectively, may have different feature spaces or different edge probability distributions, that is, *X_S_* ≠ *X_T_* or *P_S_* (*X*) ≠ *P_T_* (*X*), in addition, task *T_S_* and *T_T_* are subject to different label spaces (58).

### Statistical Analysis

Receiver operating characteristic (ROC) analysis was used to evaluate the performance of the model to identify CTC, and the area under the curve (AUC) at the 0.5 cut-off point was used to measure the prediction accuracy. At the same time, the confusion matrix was used to observe the specificity and sensitivity. All of the analyses were performed using python version 3.6.9 and “sklearn” package version 0.23.2

## Results

### Patient Characteristics

From Jan. 2017 to Jun. 2019, a total of 776 patients from Chifeng Municipal Hospital were included in this study. All the sample types were peripheral blood, and their ages ranged from 11 to 90 years old, with an average age of 65 years. Among the known cancer types, lung cancer was the most common (20.7%), followed by breast cancer and gastrointestinal cancer, and thyroid cancer was the least with only 2 cases. The clinical characteristic data of the enrolled patients were shown in **Table 1**.

**Table 1:** Summary of the general clinical information of patients.

Characteristics	No. (%) of Participants
**Age**	
0-39	30 (3.9)
40-69	313 (40.3)
>70	103 (13.3)
Unknown	330 (42.5)
**Gender**	
Male	248 (42.0)
Female	199 (25.6)
Unknown	329 (42.4)
**CTC number**	9.9(0-318)
**Cancer type**	
Lung cancer	161 (20.7)
Liver cancer	18 (2.3)
Gastrointestinal cancer	107 (13.8)
Breast cancer	91 (11.7)
Carcinoma of thyroid	2 (0.3)
NPC	30 (3.9)
Other	367 (47.3)

### Thousands of CTC and Non-CTC Images Were Segmented by openCV

The imFISH was performed on the samples from 776 patients. In order to avoid the influence of human factors, Python package *OpenCV* was used to process cell images. After the nuclear region contour was found in the blue channel (DAPI^+^), the minimum matrix coordinates of the contour were obtained, mapped to the orange channel (CEP8^+^) and segmented, and the number of centromeres was observed. If there were more than 2 centromeres, the cell was considered CTC (**Figure 2D**). Otherwise it was non-CTC (**Figures 2E, F**). Finally, we obtained 14166 images, including 694 CTC images and 13472 non-CTC images. The details of data were shown in **Table 2**, in original train set, the number of CTC and non-CTC were 555 and 10777, respectively, ensuring balanced positive and negative samples, we performed down sampling method, at last, the number of CTC and non-CTC after down sampling were 555, respectively.

**Table 2:** The number of images.

	Original			Down sampling	
	Train set	Test set	Total	Train set	Test set
No. of CTC	555	139	694	555	139
No. of Non-CTC	10777	2695	13472	555	2695
Total	11332	2834	14166	1110	2834

### The Computational Method Performed Well in Identifying CTC

**Figure 3:**
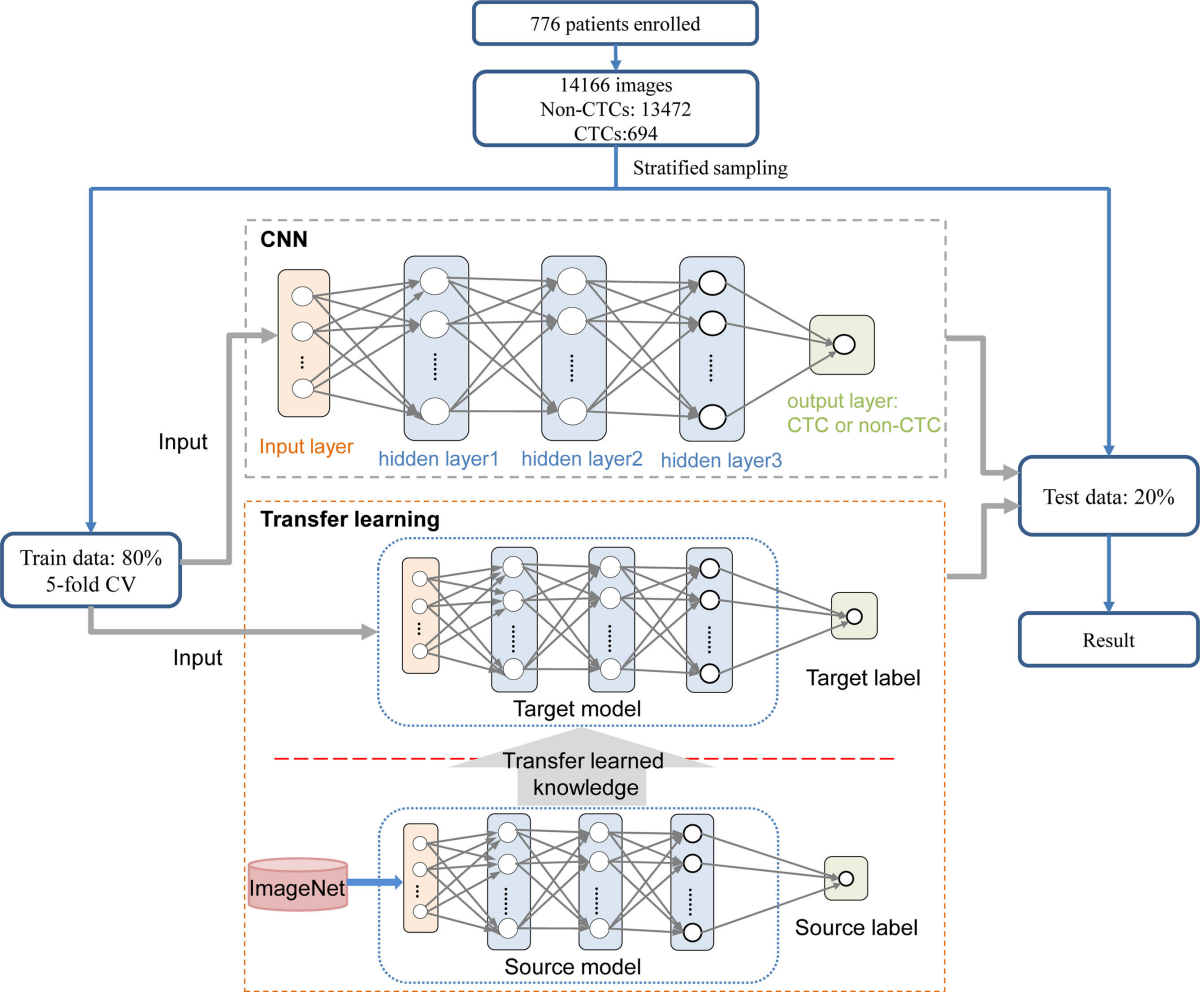
Process for identifying CTC. On the training set, the traditional CNN model and transfer learning model were respectively trained based on 5-fold CV. The transfer learning model relied on the pre-trained CNN model to realize the task of CTC recognition. The trained traditional CNN model and the transfer learning model were used to test sets and output the final results.

**Figure 4:**
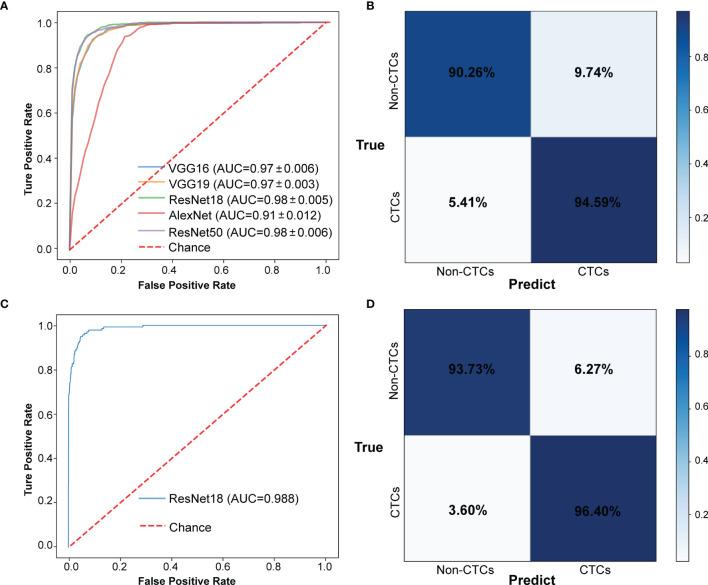
The results of CTCs identify based on traditional CNN. **(A)** ROC curve of 5-fold CV in train data set. **(B)** Confusion matrix of 5-fold CV in train data set. **(C)** ROC curve in test data set. **(D)** Confusion matrix in test data set.

**Figure 5:**
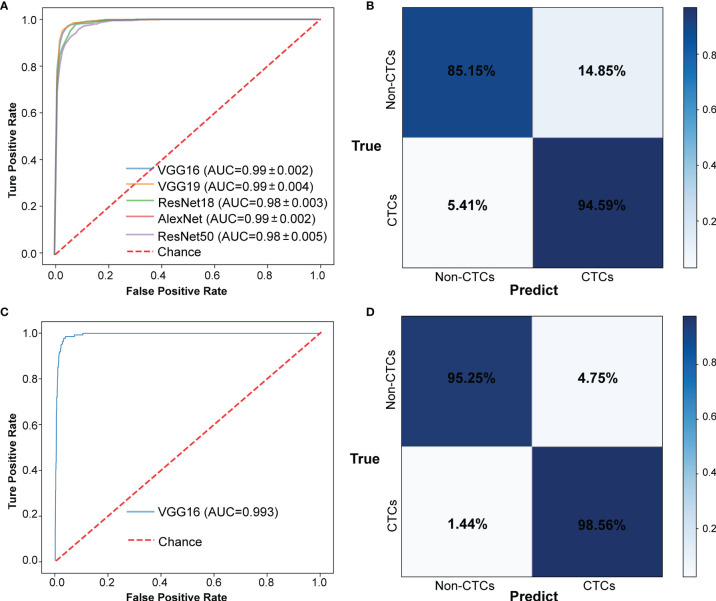
The results of CTCs identify based on transfer learning. **(A)** ROC curve of 5-fold CV in train data set. **(B)** Confusion matrix of 5-fold CV in train data set. **(C)** ROC curve in test data set. **(D)** Confusion matrix in test data set.

The CNN method was used to identify the segmented cell images. The whole process was shown in **Figure 3**. Firstly, the hierarchical sampling method was adopted for all images, 80% of the data were used for training and 20% of the data were used for testing. The CNN-based methods were used to train the model, including VGG16, VGG19, ResNet18, ResNet50, and AlexNet. Specifically, the traditional CNN model and transfer learning model were respectively trained on the training set based on 5-fold CV. The transfer learning model relied on the pre-trained CNN model to realize the task of CTC recognition. The trained traditional CNN model and the transfer learning model were used to test sets and output the final results. The results of 5-fold CV based on the trainset were shown in **Figure 4A**, the best performance was based on ResNet18 with AUC was about 0.98, in which 90.26% of non-CTCs were successfully identified, 9.74%were incorrectly identified as CTCs, while only 5.41% of CTCs are misclassified (**Figure 4B**), the sensitivity and specificity were 95.3% and 91.7% respectively. After training the model, we used ResNet18 with the best performance on the test data set, its ROC curve was shown in **Figure 4C** with AUC was 0.988, and the confusion matrix also shown that ResNet18 performed well (**Figure 4D**). In addition, in order to improve the prediction performance and save computing resources, transfer learning was also used to train the model. The results of 5-fold CV based on transfer learning in train data set was shown in **Figure 5A**, the results of confusion matrix showed that the sensitivity and specificity of transfer learning were 97.2% and 94.0% respectively (**Figure 5B**). After training the model with transfer learning, we used VGG16 with the best performance on the test data set, its AUC was 0.988 (**Figure 5C**), and the sensitivity and specificity were 98.6% and 96.5% respectively (**Figure 5D**).

**Figure 6:**
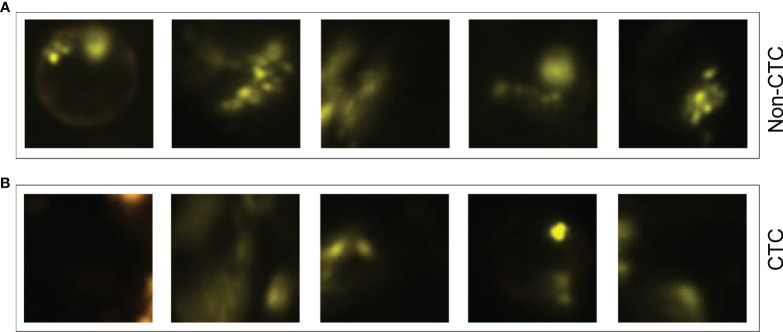
Some misjudged images. **(A)** Non-CTC images, but they were identified as CTCs. **(B)** CTC images, but they were identified as non-CTCs.

The experimental results showed that the deep learning method based on CNN can accurately identify CTC and provide a powerful reference for the prognosis of patients. In addition, we also summarized some samples that were discriminated incorrectly, such as samples that were originally non-CTC but were predicted to be CTC (**Figure 6A**), and samples that predicted CTC to be non-CTC (**Figure 6B**). The reason for the misjudgment first considers the artificial or instrumental noise in the process of negative enrichment techniques. Secondly, the exposure during the photographing process after imFISH processing resulted in us not getting the original film data. The third is that the centromere was not completely located in the nucleus due to the platform.

## Discussion

More and more evidence showed that CTCs are closely related to the prognosis of patients with advanced cancer. It has an important reference value for determining the clinical results and recurrence risk. In recent years, blood testing has been widely used to monitor the CTC response of patients with malignant tumors and evaluate the prognosis and recurrence risk of patients since its high safety and low cost. It reduces errors caused by manually setting interpretation standards and save time and labor costs. The importance of CTC rapid automatic recognition is increasing, and the research of the automatic recognition process of CTCs is also accelerating. Deep learning has been proved to be suitable for detecting CTC due to its high sensitivity and specificity in CTC counting. In addition, image interpretation using machine learning can capture important image features.

In this study, we developed a CTCs recognition method based on deep learning. After collecting the blood samples from Chifeng Municipal Hospital, we conducted CTCs enrichment and imFISH experiments on the samples, and screened the fluorescent images according to the image quality. A total of 14166 images were used for downstream analysis, including 694 CTC images and 13472 non-CTC images. 80% of the images were used for training models and 20% for test. In order to reduce the error caused by manual intervention, we used machines instead of manual screening. Firstly, images were segmented by using the Python package *openCV*, and the coordinate information of the nucleus was obtained after image preprocessing. Then, we used CNN models such as VGG16, VGG19, ResNet18, ResNet50 and AlexNet to identify CTCs. The results of 5-fold CV showed that their AUC reached 0.98, and the sensitivity and specificity were 95.3% and 91.7%,respectively. In order to overcome the shortcomings of consuming a lot of time and computing resources when developing neural networks, transfer learning was used to train the model. Finally, the AUC was improved to 0.99, and the recognition sensitivity and specificity also reached to 97.2% and 94.0% based on transfer learning.

The method of transfer learning was proposed, which can carry out image interpretation, capture important image features, reduce the errors caused by subjective factors in manual interpretation, and save computing time and computing resources. In the process of 5-fold CV, the down-sampling method was used to overcome the serious imbalance between positive samples and negative samples, and the 5-fold CV results of transfer learning shown higher sensitivity and specificity. Nevertheless, this study still has some limitations. The CTC images contained in the enrollment data do not cover the whole film, but focus on a CTC positive area under the microscope. Due to quality issues, some images in the enrollment data are abandoned. How to expand the image scope is the focus of attention in the future.

## Data Availability Statement

The datasets presented in this study can be found in online repositories. The names of the repository/repositories and accession number(s) can be found below: https://github.com/bensteven2/CTC_project.

## Ethics Statement

The studies involving human participants were reviewed and approved by Ethics Committee of Chifeng Municipal Hospital. The patients/participants provided their written informed consent to participate in this study.

## Author Contributions

FK designed the study. ZG collected, analyzed and interpreted the data, and wrote the manuscript. XL and YH performed the experiment. JW and QZ reviewed and modified the manuscript. All authors approved the final version of the manuscript.

## Funding

This work was supported by the 2020 Chifeng Natural Science Research project (grant number SZR157).

## Conflict of Interest

The authors declare that the research was conducted in the absence of any commercial or financial relationships that could be construed as a potential conflict of interest.

## Publisher’s Note

All claims expressed in this article are solely those of the authors and do not necessarily represent those of their affiliated organizations, or those of the publisher, the editors and the reviewers. Any product that may be evaluated in this article, or claim that may be made by its manufacturer, is not guaranteed or endorsed by the publisher.
